# The Gastric Vein Variants: An Evidence-Based Systematic Review of Prevalence and Clinical Considerations

**DOI:** 10.3390/jcm14113630

**Published:** 2025-05-22

**Authors:** Alejandro Bruna-Mejias, Cristian Salgado-Torres, Constanza Cáceres-Gálvez, Benjamin Rodriguez-Osorio, Mathias Orellana-Donoso, Pablo Nova-Baeza, Alejandra Suazo-Santibañez, Gustavo Oyanedel-Amaro, Juan Sanchis-Gimeno, Maria Piagkou, George Triantafyllou, Marko Konschake, Juan Jose Valenzuela-Fuenzalida

**Affiliations:** 1 Departamento de Ciencias y Geografía, Facultad de Ciencias Naturales y Exactas, Universidad de Playa Ancha, Valparaíso 2360072, Chile; alejandro.bruna@upla.cl; 2 Departamento de Morfología, Facultad de Medicina, Universidad Andrés Bello, Santiago 8370146, Chile; salgadocristian743@gmail.com (C.S.-T.); conycaceresgalvez@gmail.com (C.C.-G.); benja.biro03@gmail.com (B.R.-O.); pablo.nova@usach.cl (P.N.-B.); 3 Escuela de Medicina, Universidad Finis Terrae, Santiago 7501015, Chile; mathor94@gmail.com; 4 Faculty of Medicine and Science, Universidad San Sebastián, Santiago 8420524, Chile; 5 Faculty of Health and Social Sciences, Universidad de Las Americas, Santiago 8370040, Chile; alej.suazo@gmail.com; 6 Facultad de Ciencias de la Salud, Universidad Autónoma de Chile, Santiago 8910060, Chile; g.oyanedelamaro@gmail.com; 7 GIAVAL Research Group, Department of Anatomy and Human Embryology, Faculty of Medicine, University of Valencia, 46001 Valencia, Spain; juan.sanchis@uv.es; 8 Department of Anatomy, School of Medicine, National and Kapodistrian University of Athens, 11527 Athens, Greece; piagkoumara@gmail.com (M.P.); georgerose406@gmail.com (G.T.); 9 Neurosurgical Research Center, Petros Kokalis, National and Kapodistrian University of Athens, 11527 Athens, Greece; 10 Institute of Clinical and Functional Anatomy, Medical University Innsbruck (MUI), Müllerstrasse 59, 6020 Innsbruck, Austria; marko.konschake@i-med.ac.at

**Keywords:** variation, gastric vein, aberrant gastric vein, portal system, gastric surgery, meta-analysis, systematic review, clinical considerations

## Abstract

**Background:** The objective of the present systematic study was to analyze and characterize the gastric vein (GV) variations to understand their significance within clinical contexts, particularly in gastric and liver surgeries and managing conditions associated with the portal vein system. **Methods:** We conducted a systematic review, examining various databases, including *Medline*, *Scopus*, *Web of Science*, *Google Scholar*, *CINAHL*, *and EMBASE*, up to April 2025. Two independent authors conducted the literature search, selected pertinent studies, and extracted relevant data. The methodological quality of the studies was evaluated utilizing the Assessment Tool for Anatomical Studies (AQUA). The pooled prevalence was estimated through the application of a random effects model. **Results:** Among the 279 articles reviewed, 11 studies were ultimately incorporated into the systematic analysis, encompassing 47,993 subjects. The pooled prevalence of GV variants was determined to be 8.32%, revealing considerable heterogeneity (I^2^ = 98.92%). A subgroup analysis showed a greater prevalence of GV variants in diagnostic imaging studies than in cadaveric studies, with a higher frequency observed in males than in females. **Conclusions:** The morphological variability of the GVs holds clinical significance, as it may significantly impact the management of abdominal disorders, particularly during surgical and endovascular interventions. This study emphasizes the necessity of thorough preoperative evaluations to identify these variations, thereby minimizing surgical complications and enhancing therapeutic outcomes for patients suffering from gastric and portal vein system disorders. Integrating advanced imaging techniques into clinical practice may facilitate improved surgical and therapeutic planning.

## 1. Introduction

The gastric veins (GVs) play a crucial role in the stomach’s circulatory system by serving as the primary veins that drain blood from this organ and surrounding structures into the hepatic portal vein (HPV). The right and left gastric veins (RGV and LGV) anastomose at the lesser curvature of the stomach, draining into the lateral surface of the HPV, on both sides [1]. They are positioned and oriented parallel to the gastric arteries, right and left gastric arteries, and inferior to the lesser omentum [2,3]. The LGV, which runs alongside the LGA, passes anteriorly to the celiac artery and is positioned between the common hepatic artery and splenic artery. The LGV drains the upper section of the lesser curvature in the corpus, cardia, and lower esophagus via esophageal tributary veins. This drainage is crucial for forming gastroesophageal ulcers [4,5,6].

Other GVs and gastroepiploic veins (GEVs) originate along the greater curvature, extending to the left before draining into the SV. The right GEV originates in the pyloric region, runs along the greater curvature to the right, and empties into the superior mesenteric vein (SMV). These veins collect blood from the greater curvature and the greater omentum, channeling their flows into the portal vein system (PVS) to enable hepatic detoxification of substances absorbed from the stomach [6,7]. The present systematic review will specifically focus on analyzing the LGV and RGV, concentrating on subjects through imaging and cadaver studies, searching for subjects with and without GV variants, with the presence of symptoms being key. Clinically, the LGV and RGV are intricately associated with the HPV, establishing them as crucial pathways in portal vein (PV) circulation [7,8].

The aberrant LGV (ALGV) crosses the gastrohepatic ligament, entering the II and III hepatic segments and directly communicating with the left portal veins (LPVs) [8]. The ALGV drains the LGV and has three types: Type 1 acts as an aberrant LPV (ALPV); Type 2 partially anastomoses intrahepatically to the LPV; Type 3 completely anastomoses intrahepatically with the LPV [9,10]. The LGV drainage may vary, typically into the HPV and the SV or intrahepatically into the PVS [9,10,11]. Following Lee’s classification (2019), the LGV has four types: (1) Type I crosses the common hepatic artery into the PVS; (2) Type II drains anteriorly to the celiac artery; (3) Type III crosses the splenic artery to drain into the HPV; (4) Type IV drains directly into the liver or proximal PV. The ARGV, extending about 6 cm through the hepatic ligament, aligns with the LPV [12]. ARGVs that drain into the second liver or peripheral PV branch have a prevalence of approximately 1.5%, while ALGV is a rare variant with a prevalence of less than 1.0% [13].

These morphologies have crucial clinical implications. Variants like ALGV help reduce gastric variceal bleeding in cirrhotic patients [10]. ARGV maintains a hepatic flow, aiding in the placement of a transjugular intrahepatic portosystemic shunt (TIPS) when HPV recanalization is impossible [14]. LGV can lead to atrophy in the second hepatic segment and facilitate metastasis of gastric tumors to the left hepatic lobe [15]. Preoperative analysis of LGV is essential to identify its location and reduce surgical risks, while understanding interportal venous communication is vital for treating esophageal varices and planning embolization [16]. The current study aims to explore GV morphological variants using systematic and statistical methods to assess the occurrence of clinically significant variants. Abdominal vein variations can be very complex, and if not identified quickly, they may hinder diagnostic and surgical processes. Therefore, detecting possible morphological variants through imaging is crucial to facilitate the precise diagnosis and management of conditions or surgical issues related to the GV and adjacent regions. Additionally, when considering conditions like portal hypertension, a detailed knowledge of the stomach’s vascular anatomy is vital to mitigate complications associated with heightened blood flow through the PV. Ultimately, we propose that diagnostic studies aimed at improving our understanding of these variants and their clinical consequences could significantly enhance our anatomical and clinical insights into the subject.

## 2. Materials and Methods

### 2.1. Protocol and Registration

We were guided by the PRISMA statement [17] in carrying out this evidence-based systematic review. The registration number in the Systematic Reviews Registry (PROSPERO) is CRD42024574548 (9 August 2024).

### 2.2. Electronic Search

We explored various databases in January to identify the most relevant studies for our research question. These included *MEDLINE* (via *PubMed*), *Google Scholar*, *Web of Science (WOS)*, *CINAHL*, *EMBASE*, and *Scopus*, covering the timeframe from their inception until July 2024. Our search strategy utilized a blend of terms: “gastric veins” (Not Mesh), “gastric drainage” (Not Mesh), “portal system” (Not Mesh), “variations gastric veins” (Not Mesh), “clinical anatomy” (Not Mesh), and “anatomical variation” (Not Mesh), incorporating the Boolean operators AND, OR, and NOT (Supplementary Table S1).

### 2.3. Eligibility Criteria

The eligibility criteria for this review included studies that examined variations in the morphology of the GV and their correlation with specific clinical conditions. Studies were deemed eligible if they met the following requirements: (1) Sample: dissections or images that demonstrated GV variations; (2) Results: reported prevalence of subjects with GV variants and their associations with abdominal pathologies; (3) Studies: this systematic review considered research articles of both retrospective and prospective observational designs, published in English in peer-reviewed journals, and indexed in relevant databases. To determine exclusion criteria, we applied the following filters: (1) Sample: studies conducted on animals; (2) studies that focused on variants unrelated to the hepatic region or its drainage area; (3) studies that included letters to the editor or commentary.

### 2.4. Study Selection

Three authors independently analyzed the studies to select them thoroughly. Initially, two authors (*Valenzuela JJ and Salgado C*) reviewed the titles and abstracts of the references retrieved from the database searches. For the selected studies, we obtained the full text of the references that any authors deemed potentially relevant. A third reviewer (*Caceres C*) was involved if a consensus could not be reached. To assess the reliability and the risk of bias among the observers, we performed the kappa agreement test between the authors, which yielded a score of 0.80, interpreted as indicating good agreement (Supplementary Table S2).

### 2.5. Data Collection Process

Two authors (*Nova P and Orellana M*) independently extracted data on the outcomes of each study. The data extracted from the included studies were as follows: (a) authors and year of publication, (b) total sample size and age, (c) prevalence, (d) variant characteristics, (e) regional geography, (f) sex of the sample, and (g) clinical considerations.

### 2.6. Assessment of the Methodological Quality of the Included Studies

To assess the bias in the included studies, we utilized the verification table for anatomical studies (AQUA) developed by the International Working Group on Evidence-Based Anatomy (IEBA) (Tomaszewski et al., 2016) [18]. Two reviewers (*Valenzuela JJ and Nova P*) independently examined the five domains outlined by the AQUA tool and then collaborated to build the table and construct the bias graph.

### 2.7. Statistical Methods

The data was analyzed using R statistical software (version 2025.05.0+496) (https://posit.co/download/rstudio-desktop/, accessed on 1 January 2025) to determine the prevalence of GV morphological variants. Summary data were combined using the DerSimonian–Laird model and a Freeman–Tukey double arcsine transformation. A random effects model was employed due to the high heterogeneity in the prevalence of GV variants. The heterogeneity among the included studies was assessed using the chi^2^ test and the I^2^ statistic. A *p*-value of 0.10 for the chi^2^ test was deemed significant, following Cochrane collaboration guidelines [18]. The I^2^ statistic values were interpreted within a 95% confidence interval (CI) as follows: 0–40% indicated no significant heterogeneity, 30–60% suggested moderate heterogeneity, 50–90% indicated substantial heterogeneity, and 75–100% reflected considerable heterogeneity [19]. To investigate small study effects (*where smaller studies may yield different results compared to larger ones*), a DOI plot incorporating the LFK index was produced [20,21].

### 2.8. Subgroup Analysis

We performed the same statistical analyses across each subgroup to reduce biases that might result in underestimating or overestimating the subgroup results. We also included prevalence rates for each group and qualitative assessments of their clinical implications. Ultimately, we categorized the subgroups into three classifications: imaging studies, patients, and cadaveric specimens, allowing for a thorough individual analysis for each category.

## 3. Results

### 3.1. Selection of the Articles

**Figure 1:**
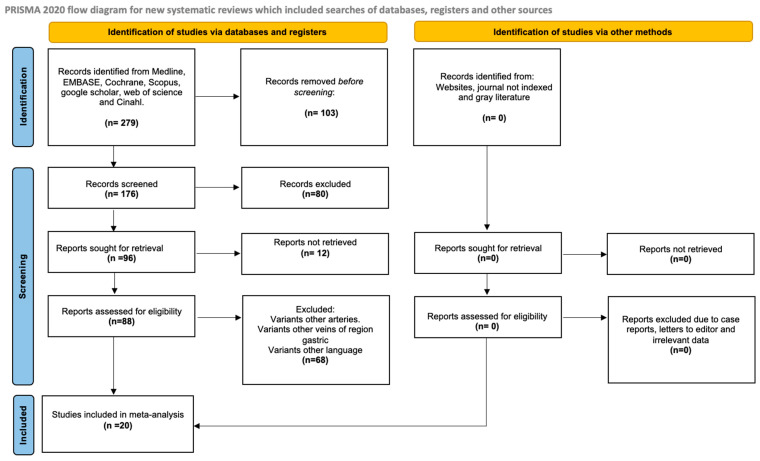
Search diagram of the methodology process.

The search yielded 279 articles from various databases, aligning with our research team’s criteria and search terms. The filtration process focused on these articles’ titles and/or abstracts. From the initial pool of 31 articles included [8,9,10,11,12,13,14,16,22,23,24,25,26,27,28,29,30,31,32,33,34,35,36,37,38,39,40,41,42], 11 studies were selected for inclusion [9,10,11,13,29,30,31,34,38,40,43]. These articles were chosen for their comprehensive survey of the sample, detailed statistical data for each variant, and transparent methodology (Figure 1).

### 3.2. Characteristics of the Included Studies

Thirty-one studies included 47,993 subjects. Eleven studies contributed to the calculated prevalence. Geographic distribution: 17 studies from Asia, 11 from Europe, 1 from Oceania, 2 from the Americas, and 0 from Africa. Sex was reported for 3857 subjects: 2618 men and 1239 women. The mean age was 53.8 years (Table 1 and Table 2).

### 3.3. Description of the Variants

GVs, especially LGV and RGV, are crucial for stomach venous drainage. The LGV collects blood from the upper stomach, including the cardia and body, ascends along the lesser curvature, and drains into the HPV. It runs near the LGA and connects to the esophagus and pylorus. The RGV drains blood from the lower stomach, including the pyloric antrum. It parallels the LGV and the lesser curvature and drains into the HPV, accompanying the right gastric artery. Understanding these veins’ pathways and significance is key to their clinical relevance.

**Figure 2:**
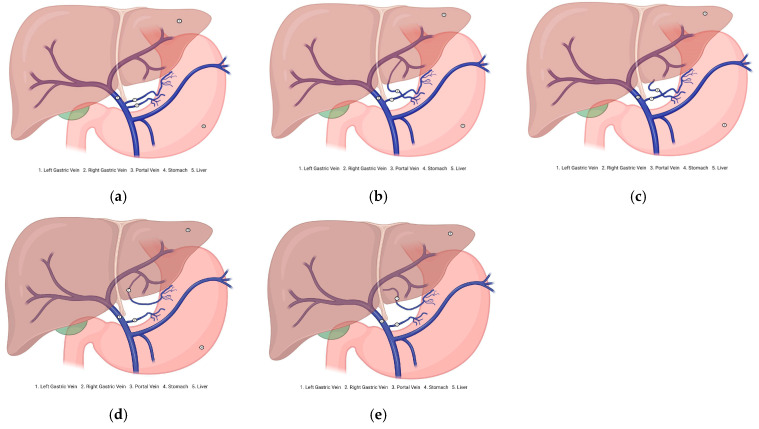
Diagram of the gastric vein morphological variants. (**a**) Normal venous drainage of the stomach. (**b**) Aberrant right gastric vein drains into the portal vein branch. (**c**) Aberrant right gastric vein draining into the hepatic parenchyma. (**d**) Aberrant left gastric vein draining into the portal vein branch. (**e**) Aberrant left gastric vein draining into the hepatic parenchyma.

Aberrant gastric veins (AGVs) may correspond to atypical venous vessels or morphological variants of the stomach that diverge from the standard venous paths. They can connect unusually with veins, such as the SV or mesenteric vein (MSV), thereby altering drainage patterns. For instance, ALGVs drain into branches of the HPV instead of directly, a pattern that applies to RGVs (Figure 2).

AGVs can create clinical challenges. Their unexpected locations complicate surgeries, such as gastrectomy and liver resection, where accurate anatomical knowledge is crucial. In portal hypertension, these veins can increase the risk of varices by acting as collateral pathways. Recognizing variations in imaging studies, including computed tomography angiography (CTA) and magnetic resonance angiography (MRA), is crucial for anticipating complications and enhancing patient outcomes. Furthermore, since GV morphological variations may also impact the effectiveness of treatments such as TIPS, a thorough understanding of individual venous anatomy is essential for customizing interventions. Consequently, comprehensive studies enhance the anatomical knowledge base and improve clinical practice by informing preoperative planning and management strategies. Therefore, identifying and documenting these variants in the present research can have significant implications for surgical safety and the management of gastrointestinal conditions.

#### 3.3.1. Prevalence and Subgroups Analyzed

**Figure 3:**
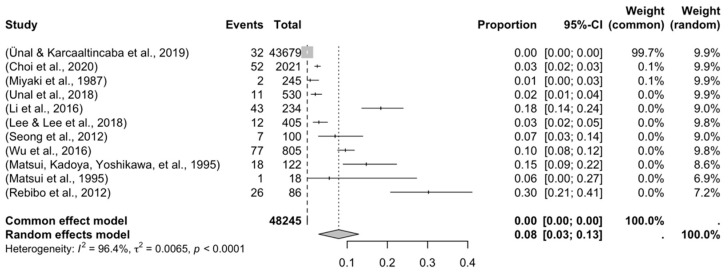
Forest plot of the articles included in the meta-analysis.

**Figure 4:**
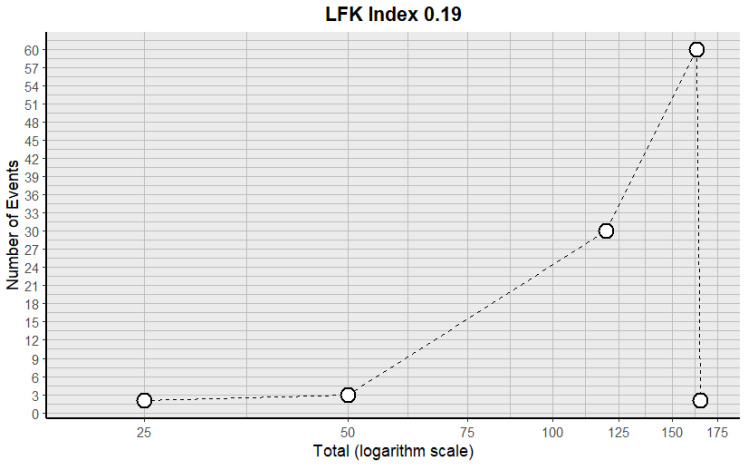
DOI plot with the LFK index for the included studies.

Four forest plots were created to calculate the prevalence of GV variants in the eleven studies [9,10,11,13,29,30,31,34,38,40,43] (Table 3). A prevalence of 8.32% (CI: 3.12–13.17) was estimated. The samples’ heterogeneity was 98.92%, indicating high variability, with a quite heterogeneous sample (Figure 3). The detection of publication bias was quantified using a DOI graph to visualize the asymmetry, with the LFK index showing a non-significant publication bias of 0.19 (Figure 4).

Regarding the subgroup analysis, we grouped studies with a prevalence of no more than 50%. The first subgroup consisted of cadavers and images. In the cadaver subgroup, one study was included [34] with a prevalence of 0.082%. Among the imaging studies, 10 were included [9,10,11,13,29,30,31,34,38,40,43], revealing a prevalence of 8.18% (CI: 3.49–12.91) and a heterogeneity of 89.11%. This subgroup analysis demonstrated a significant difference, indicating a greater presence of diagnostic images (*p* = 0.0001).

The *second subgroup analysis* was for the continents from which the included studies were conducted. From Asia, eight studies were included [11,13,29,30,31,34,40,43], which presented a prevalence of 6.37% (CI: 4.19–8.98) and a heterogeneity of 88.12%. From Europe, three studies were included [9,10,38], presenting a prevalence of 2.11% (CI: 0.77-3.99) and a heterogeneity of 91.35%. Prevalence articles from America, Oceania, and Africa were not included. For this subgroup analysis, there was a significant difference in the presence of studies from Asia compared to Europe (*p* = 0.032).

*Other subgroup analyses* were performed on the sex of the subjects included. Seven studies showed males with the variant [11,13,29,30,31,40,43], which presented a prevalence of GV variants of 5.29% (CI 3.12–9.33); the heterogeneity of this comparison was 77.1%. Meanwhile, seven studies reported females with the variant [11,13,29,30,31,40,43], presenting a prevalence of 2.43% (CI 1.10–4.13) and a heterogeneity of 84.12%. For this subgroup analysis, there was a significant difference in favor of the greater presence of GV variants in males than in females (*p* = 0.024).

*Further subgroup analyses were performed for the left gastric artery (LGA) and right gastric artery (RGA) variants*. Ten studies showed LGV variants [9,10,11,13,29,30,31,38,40,43], with a prevalence of 8.18% (CI 7.01–10.12); the heterogeneity of this comparison was 77.1%. Meanwhile, three studies reported RGV variants [9,31,43], presenting a prevalence of 3.29% (CI 2.21–4.13) and a heterogeneity of 88.12%. For this subgroup analysis, there was a significant difference in favor of the greater presence of the LGV variants than the RGV variants (*p* = 0.012) (Table 4).

#### 3.3.2. Risk of Bias in the Included Articles

**Figure 5:**
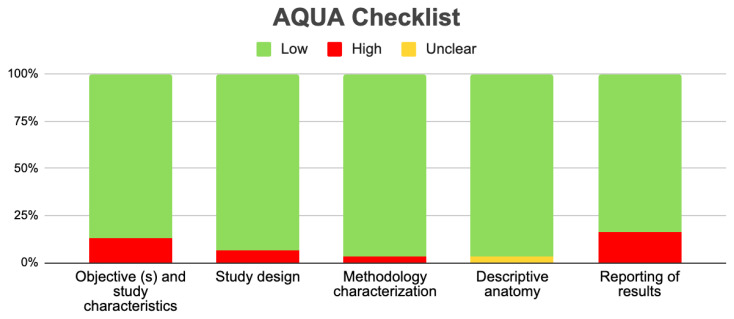
Aqua chart checklist of the quality of the included studies.

Applying the AQUA checklist to the three reviewed studies allowed us to assess potential biases across the five domains: *selection*, *performance*, *detection*, *attrition*, *and reporting bias*. The low risk of bias suggests that these studies are reliable in providing accurate and clinically relevant information about GV morphological variations. The results are strong, enabling confident conclusions about the clinical implications of the identified variants. Figure 5 visually summarizes the assessment, highlighting the strengths of the included evidence in our evidence-based systematic review. We found a significant level of heterogeneity in the studies, primarily due to the variability in total sample sizes among studies that compare different GV variants. To address this issue, a random statistical model was employed. Additionally, a subgroup analysis was conducted, providing a more detailed examination with reduced heterogeneity. Therefore, while our results are robust, the high heterogeneity warrants caution in their interpretation.

## 4. Discussion

This systematic review investigated the GV morphological variants, with a primary emphasis on drainage variants and their relationship to the stomach. Our main finding reveals that, although these GV variants are rare in the population, they show considerable differences in their descriptions and drainage areas, which frequently complicates their understanding. The unfamiliarity with these variants can lead to difficulties in diagnosing various conditions affecting the stomach or adjacent structures, as well as intraoperative complications in different abdominal regions. Moreover, these morphological variants have been linked to a heightened risk of alterations in the venous drainage of tumors in the pyloric region and the lesser curvature of the stomach, highlighting the importance of understanding these variations.

The present systematic review compiles evidence on GV morphological variants, as no similar studies exist. Previous reviews mentioned GV variants superficially or indirectly. Frey (2022) [8] discussed the aspects of ALGV that are important for surgeons but lacked an in-depth examination of morphological variants. Meanwhile, Stefura (2018) [44] highlighted the venous trunk of Henle in portal circulation but only briefly touched on GV variants, underlining the novelty of this research. AGVs deviate from the stomach’s typical drainage, connecting abnormally to other veins, such as the SV or SMV. In portal hypertension, the AGVs can become prominent, increasing the risk of varicose veins and bleeding during abdominal surgery [27].

Regarding the quantitative results found in the present review, we discovered that the prevalence was linked to the description of a rare variant in the gastric region, defined as occurring in no more than 10% of the population. The studies analyzed for prevalence showed high heterogeneity; therefore, the reported results should be interpreted cautiously. On another note, we examined the studies that presented a quantifiable prevalence through subgroups, determining whether the sample studied consisted of cadavers or a collection of patient images. A significant difference was noted in favor of diagnosing or recognizing the variants through images, suggesting that this variant could be identified at some point in life due to a gastric alteration or surgery in the area. Regarding whether this variant produces symptoms on its own, we found no evidence to support this theory, leading us to believe that the diagnosis or discovery of this variant is random. The following subgroup analysis was by geographic region, including samples from Asia, Europe, America, Africa, or Oceania, where a statistically significant difference was found among the subjects from Asia. Two possible explanations could account for this finding: first, more studies have been conducted in that region; second, this variant may be more commonly associated with Asian populations. However, we could not find sufficient evidence to support these claims. In conclusion, our analysis indicates a significantly higher prevalence of GV variants among males. However, we lacked evidence to explain or substantiate this relationship. While these findings contribute to understanding the characteristics of GV variants, they should be interpreted cautiously, considering that some studies report a high overall prevalence. Additionally, this systematic review identifies several GV variants that are associated with significant clinical implications, particularly in abdominal conditions requiring surgical intervention [15]. These variants may facilitate surgical procedures, enhance venous drainage, or improve blood flow under certain physiological conditions. Such findings highlight the necessity of a thorough understanding of GV anatomy for clinicians, especially in surgical contexts where awareness of these variants can lead to better outcomes and fewer complications. Recognizing and documenting these morphological variations can also assist in preoperative planning and managing issues like portal hypertension [45]. According to Unal’s (2019) [9] classification, ALGV Type III signifies a complete anastomosis between the ALGV and the LPV. This variant preserves hepatic flow due to the anastomosis in patients with main HPV thrombosis, serving as a decompression pathway that significantly reduces the development and severity of extensive gastric variceal bleeding in cirrhotic patients with hypertensive gastropathy [27]. In instances of main HPV thrombosis, the ARGV can also sustain adequate hepatic flow, because the venous drainage of the lesser curvature of the stomach can operate independently of the main HPV, maintaining the patency of the intrahepatic portal system [24]. In the context of significant clinical procedures, portosystemic shunts, which can be total or intrahepatic, are employed. These shunts have defined indications and effects and are crucial in minimizing the risk of variceal bleeding, a potentially life-threatening complication in patients with portal hypertension. Total portosystemic shunts redirect all portal circulation to the vena cava, thus eliminating hepatoportal flow, which may affect liver function and increase the risk of encephalopathy [46]. The transjugular intrahepatic portosystemic shunt (TIPS) procedure, performed by interventional radiology, aims to lower the hepatic venous pressure gradient to below 12 mmHg. Using ultrasound (US), a catheter is inserted through the right internal jugular vein into the RHV, connecting it with the intrahepatic PV. The tract is dilated with an angioplasty balloon, and a stent is placed, resulting in a reduced portal pressure gradient and decreased blood flow to collaterals like esophageal and gastric varices [46]. The ARGV acts as an aberrant PV (APV) for TIPS patients whose main PV trunks cannot be recanalized. In cases of HPV thrombosis, when recanalization fails, this variant may be the only pathway for TIPS placement [14].

In addition, 14 studies were identified that described pathological considerations regarding GV variants [8,10,11,12,13,22,25,26,31,33,37,41,42]. Variceal bleeding is a common complication in patients with portal hypertension, directly linked to the LGV, as it is the primary source of blood supply for esophagogastric varices [11]. Such bleeding is more frequent in patients whose LGV venous diameter exceeds 5–6 mm, a parameter indicative of portal hypertension [11]. Furthermore, in 33.3% of patients with ALGV, atrophy of the second hepatic segment is observed, producing pseudolesions due to the imbalance in hepatic blood flow [8]. Additionally, ALGV provides a direct route for the periportal spread of gastric tumors through the gastrohepatic ligament, facilitating metastasis in the left hepatic lobe. An even lower survival rate is estimated in cases of gastric cancer with venous invasion facilitated by this variant [8]. The presence of ALGV was associated with HPV gas affecting only the left hepatic lobe due to drainage from the variant into the LPV in a patient with gastric pneumatosis secondary to a hiatal hernia [33]. Therefore, identifying variants in GV drainage through imaging is essential to prevent invasive procedures, enhance the understanding of related hepatic pseudolesions, and improve the monitoring of gastric cancer, particularly given the presence of these variants in cases of venous–hepatic metastasis [42].

Additionally, six imaging studies were identified [23,34,35,36,39,47]. The US study of the LGV termination defines its anatomical relationship with the adjacent vessels and drainage [34]. The CTA study of the ARGV is relevant when evaluating hepatic metastatic diseases, since hepatic vascular variants can be confused with more serious pathologies [47]. Three-dimensional CTA is essential for visualizing the peri-gastric anatomy preoperatively. Additionally, knowing about vascular anomalies, such as ALGV, in the liver vasculature is crucial for avoiding unnecessary invasive procedures and linking them to pseudolesions in the liver parenchyma [23]. Therefore, accurate imaging analysis is crucial for the differential diagnosis of different liver conditions. To optimize the clinical outcomes and reduce the perioperative risk, the integration of advanced imaging techniques such as CTA and magnetic resonance angiography (MRA) is strongly recommended for preoperative and diagnostic evaluation of gastric venous anatomy. CTA offers high spatial resolution and rapid acquisition, making it ideal for delineating venous drainage patterns, identifying anomalous venous pathways, and assessing their relationship with adjacent organs and vessels, particularly in patients with portal hypertension, hepatic malignancies, or those undergoing gastrectomy or bariatric procedures. In contrast, MRA, while slightly less spatially precise, provides excellent soft tissue contrast without ionizing radiation or iodinated contrast, which is advantageous in patients with renal dysfunction or requiring serial assessments. It is beneficial in assessing the flow characteristics, collateral formation, and chronic changes in venous morphology. Clinicians and surgeons should implement routine pre-interventional CTA or MRA in high-risk cases, including patients with suspected venous anomalies, those scheduled for laparoscopic gastric surgery, and individuals with cirrhosis or portal vein thrombosis, to enhance the anatomical precision and guide the intraoperative navigation. Moreover, adopting 3D reconstruction and vessel segmentation software (version 11.1.2) can facilitate multidisciplinary planning, enabling safer ligation, anastomosis, or shunt placement and reducing the likelihood of inadvertent vascular injury. Finally, seven studies have been identified that describe key surgical considerations in the management of patients with GV variants [14,16,28,29,31,38]. In this context, the gastrectomy procedure, primarily performed laparoscopically, is categorized into three types: *atypical*, *subtotal*, *and total gastrectomy*. Atypical gastrectomy is for resecting gastrointestinal stromal tumors with specific criteria. Subtotal gastrectomy partially removes the stomach, depending on pathology, possibly taking one-half, two-thirds, or three-fifths of it. An anastomosis connects the gastric stump to the duodenum (Pean Billroth I) or jejunum (Billroth II) to restore digestive continuity. Total gastrectomy removes the stomach and connects the jejunum with the esophagus or a Roux-en-Y loop [48]. The surgical technique adapts to the patient’s characteristics and the surgical team’s preferences. The procedure starts with the patient supine in the anti-Trendelenburg position, establishing pneumoperitoneum and placing five trocars, typically in the right and left anterior axillary line, left flank, and subxiphoid region. Next, the greater curvature is dissected, ligating the gastric vessels while preserving the gastroepiploic vessels for greater omentum irrigation. The lesser omentum is then sectioned, and the pyloric artery is ligated about 2–3 cm from the duodenum to enable the release and sectioning of the duodenal bulb, which is subsequently closed [49,50]. Depending on the objectives, gastrectomy can be partial or total, involving gastrojejunal anastomosis and stomach resection. The excised gastric portion is often not removed during gastric bypass; when needed, it is extracted via the umbilical route [48]. In the Type II variant [29], where the LGV runs anterior to the CA draining into the HPV, LGV injury during laparoscopic gastrectomy is 5. 5.8%, significantly lower than in variants I and III. Type II LGV is easily detectable without complete lymph node dissection. Injuries to LGVs with anterior drainage (Types Ia, II, and IIIa) were less common than with posterior drainage (Types Ip and IIIp) [29]. If the LGV is not visible anteriorly, surgeons must carefully avoid injuring it among the CHA or SA lymph nodes during standard gastrectomy, which involves re-sectioning at least two-thirds of the stomach [29]. In cases of ALGV and ALHA, the ALGV is divided at the root while preserving the ALHA, with only the branches extending into the stomach being divided to prioritize patient safety [28]. Pancreatectomy treats tumors in the pancreas or lower common bile duct, performed openly or laparoscopically, depending on the disease extent, involving resections of various pancreatic portions [51]. The open technique uses a supraumbilical incision to access the pancreas and surrounding structures, starting with greater omentum dissection, which separates the pancreatic isthmus from the portal vein. The trunk of Henle is ligated to prevent hemorrhage. Cephalic or total pancreatectomy requires cholecystectomy and bile duct mobilization to ensure adequate resection and reduce complications [51]. Confirming LGV anatomy is crucial before pancreatectomy, as damage can lead to ischemic gastric complications requiring additional gastrectomy. A portal phase abdominal CT scan with contrast visualizes the LGV and variants [38]. Pancreatectomy is a surgical procedure often performed for tumor pathologies affecting the pancreas or the lower common bile duct. It can be executed using open or laparoscopic techniques involving the resection of various portions of the pancreas: cephalic, left, medial, or total, depending on the extent of the disease [51]. The open technique involves a supraumbilical incision to access the pancreas and nearby structures, including the trunk of Henle, formed by the right gastroepiploic, middle colic, and inferior pancreaticoduodenal veins. Surgery begins with cutting the greater omentum outside the GEVs, freeing the pancreatic isthmus from the PV. Next, the trunk of Henle is ligated to prevent bleeding. For cephalic or total pancreatectomy, cholecystectomy and bile duct release are necessary for proper resection and to avoid complications [51]. Establishing the anatomy of the LGV is crucial before a pancreatectomy, as damage to this vein can lead to ischemic gastric complications and possible gastrectomy. Thus, a portal phase abdominal CT scan with contrast helps visualize the LGV and its variants [38]. When the LGV terminates at the SV or between the HPV and SV confluence, dissection must be cautious; during pancreatectomy, ligate the SV above its junction with the LGV [38]. In cases of venous resection, care should be taken to ensure that the LGV is connected to the HPV axis (either by repositioning the SV or by anastomosis directly to the HPV), as when the LGV terminates at the HPV, there is little risk of damage during a pancreatectomy [38] (Table 5 and Table 6).

GV variants, particularly aberrant drainage patterns involving the LGV, short GVs, or posterior GVs, can significantly influence portal hemodynamics and predispose individuals toward various clinical complications. For instance, in patients with portal hypertension, atypical drainage of the LGV directly into the systemic circulation or the azygos system can serve as a collateral channel, exacerbating the formation of esophageal or gastric varices and increasing the risk of life-threatening upper gastrointestinal bleeding. Similarly, variations in which GVs drain into the SV or inferior mesenteric veins may alter regional pressure gradients, contributing to localized portal hypertensive gastropathy or even segmental colitis resulting from venous congestion. Furthermore, in surgical contexts, unrecognized variants may lead to accidental ligation or injury of ectopic GVs, resulting in intraoperative hemorrhage or postoperative ischemia of gastric tissue. These pathophysiological cascades underscore the clinical importance of preoperative imaging and thorough venous mapping in patients undergoing upper abdominal surgeries or interventional procedures for portal hypertension [52,53,54,55,56,57,58,59,60,61].

Our results aligned with past studies highlighting the significance of venous variants in gastrointestinal complications. Our analysis offers a detailed characterization of these variants and their clinical correlations, showcasing a comprehensive systematic review. Detecting variants via imaging is vital for diagnosing and managing related pathologies. Therefore, we advocate for the use of advanced imaging techniques in patients with gastrointestinal complications. Previous studies by Miyaki (1987) and Yamagami (1999) [34,47] emphasized the roles of US and CTA in assessing GV drainage, especially for aberrant variants. Future research ought to pursue longitudinal studies examining the long-term effects of these variants on gastrointestinal health, in conjunction with more extensive studies utilizing standardized classification methods. This study emphasizes the significance of GV variants in clinical complications, advocating for their consideration in clinical practice to enhance surgical planning and management for patients at risk of portal hypertension, thereby ultimately improving clinical and surgical outcomes.

## 5. Conclusions

Variants of abdominal blood vessels can be remarkably intricate, and if not identified promptly, they may complicate diagnostic and surgical procedures. Consequently, it is imperative to recognize these variants through diagnostic or pre-surgical imaging to ensure the accurate diagnosis and management of conditions or surgical complications that may involve the GV and adjacent areas. Furthermore, in the context of pathologies such as portal hypertension, a comprehensive understanding of the vascular anatomy of the stomach is essential to prevent complications that may arise from increased blood flow through the PV. To optimize the outcomes and reduce the perioperative risk, integrating advanced imaging techniques like CTA and magnetic resonance angiography (MRA) is recommended for the preoperative evaluation of gastric venous anatomy. Ultimately, we believe that diagnostic studies focused on enhancing our understanding of these variants and their clinical implications could significantly contribute to more comprehensive anatomical and clinical knowledge of the matter.

## Figures and Tables

**Table 1:** Characteristics of the included studies (ALGV—aberrant left gastric vein, CT—computed tomography, and CTA—computed tomography angiography).

Author (s)	Year	Geographic Region	Type Study/Sample (n)	Mean Age (Range)/Sex	GV Variants’ Classification
Ünal & Karcaaltincaba et al.	2019	Turkey	Abdominal CT scans/43,679 subjects	Not specified	32 subjects with ALGV: **Type 1:** 22, **Type 2:** 3, and **Type 3:** 7
Li et al.	2016	China	upper abdomen MDCTA/234 patients	49.9 (18–81)/123 M and 111 F	**Type 1:** 108 (46.15%), **Type 2:** 72% (30.77%), **Type 3:** 34 (14.53%), and **Type 4:** 9 (3.85%)
Lee & Lee et al.	2018	South Korea	Gastrectomy for gastric AdenoCa/ 405 patients’ laparoscopic	64 (30-92)/259 M and 146 F	**Ia:** 3.0% (n = 12), **Ip:** 48.1% (n = 195), **II:** 30.0% (n = 121), **IIIa:** 12.3% (n = 50), **IIIp:** 5.7% (n = 23) and **IV:** 1.0% (n = 4)
Kuwada et al.	2015	Japan	Preoperative Abdominal CT/1 Case	60/F	1/1
Ishigami et al.	2004	USA	CTA/1 patient	20/M	1/1
Frey et al.	2022	France	preoperative CT/3 patients	49/M, 60/M, 59/M	3/3
Deneve et al.	2003	France	Autopsy/1 case	64/F	1/1
Caty et al.	2004	France	Autopsy/1 case	46/M	1/1
Choi et al.	2020	South Korea	CT/2021 patients	62 (20–96)/1572 M and 449 F	**Type 1:** 31, **Type 2:** 21
Arhire et al.	2023	Romania	Ultrasound and CT/2 patients	59/M, 63/M	1/1, 1/1
Mittal et al.	2015	India	Ultrasound and CT/1 patient	74/M	**Type 1:** 1
Miyaki et al.	1987	Japan	dissection/245 patients	Not specified	**Type 1:** 1, **Type 2:** 1
Ohkubo et al.	2000	Japan	Dissection/1 patient	51/F	**Type 1:** 1
Alfaro et al.	2023	Colombia	CT/3 patients	62/F, 46/F, and 50/M	**Type 1:** 1, **Type 2:** 1, and **Type 3:** 1
Seong et al.	2012	Korea	Gastric arteriography/100 patients	56.2/73 M and 27 F	**Type 1:** 9, **Type 2:** 12 (2a: 3, 2b: 9), **Type 3:** 38 (3a: 22, 3b: 16), and **Type 4:** 7
Unal et al.	2015	Turkey	Not specified	Not specified	Types 1, 2, and 3
Unal et al.	2018	Turkey	CT/530 patients Venous invasion in 11 patients	60/6 M and 5 F	**Type 1:** 3 **Type 2:** 2, **Type 3:** 2, **Type 4:** 2, and **Type 5:** 2
Wu et al.	2016	China	CT/805 patients	53/432 M and 393 F	**Type 1:** 401, **Type 2:** 166, **Type 3:** 161, **Type 4:** 59, **Type 5:** 12, **Type 6:** 6, and **Type 7:** 0
Yamagami et al.	1999	Japan	CTA/1 patient	50/M	**Type 1:** 1
Muñoz & Fraum et al.	2023	United States	PSMA-PET/CT and MRI/1 patient	53/M	**Type 1:** 1
Bezzi et al.	1995	Italy	transjugular intrahepatic portosystemic shunt/2 patients	50/M, 58/M	2/2
Deger & Bozer et al.	2023	Turkey	Abdominal US/1 patient	49/M	1/1
Gabata et al.	1997	Japan	/17 patients with fatty liver	56 (40 to 72 years)/12 M and 5 F	5/17
Hiwatashi et al.	1999	Japan	Angiography/1 patient	54/M	1/1
Matsui et al.	1994	Japan	CT and CTAP (arterial portography)/**Group A:** 22 patients with pseudolesions in segment IV. **Group B:** 100 random patients without pseudolesions	38–68 years/**Group A:** 15 M, 7 F 35–83 years/**Group B:** 67 M, 33 F	AGVD18 in group A AGVD 0/100 patients in group B
Matsui, Kadoya, Yoshikawa, et al.	1995	Japan	CT and CTAP/18 patients	38–68 years/11M and 7 F	17/18 and 1/18
Matsui et al.	1995	Japan	CTAP and/or hepatic arteriography/17 patients	43–73 years/8M, and 9 F	7/17
Natsume et al.	2010	Japan	64-row MDCT (portal venography)/126 patients	66/-	In 52 patients (44%), LCV flowed into the PV, 44 (37%) into the SV 22 (19%) into the junction of these two veins
Terayama et al.	2004	Japan	post-contrast helical CT/1 patient	36/M	1/1
Roi et al.	1993	Wales	Abdomen US/86 patients	3–83 years/24 M and 62 F	Drain **Type 1:** 26 (30%), **Type 2:** 28 (33%), **Type 3:** 32 (37%) Course (n = 83) **Type 1A:** 3, **Type 1P:** 21, **Type 2A:** 10, **Type 2P:** 17, **Type 3A:** 26 and **Type 3P:** 6
Rebibo et al.	2012	France	CT/86 patients	Does not refer	**Type A:** 65%, **Type B:** 4.7%, and **Type C:** 30.3%

**Table 2:** Characteristics of the variants and clinical implications (APV = aberrant portal vein, PV = portal vein, LGV—left gastric vein, SV—splenic vein, and ALGV—aberrant left gastric vein).

Author (s)	Year	Characteristics	Variant Type	Clinical Implications
Ünal & Karcaaltincaba et al.	2019	**Type 1:** Vein acting as a pure aberrant portal vein (APV) branching through the parenchyma. **Type 2:** The vein has a parenchymatous distribution and anastomosis to the left intrahepatic PV. **Type 3:** The vein has anastomosis to the left intrahepatic PV branch.	Drain	Hyperdensity at the posterior of segments II and III in type 1 (n = 20/22) and type 2 (n = 2/3) ALGV patients shows fat sparing from the third inflow effect. The ALGV maintains specific blood flow to liver segments during thrombosis.
Li et al.	2016	**Type 1:** The LGV originated from the PV. **Type 2:** The LGV originated from the SV. **Type 3:** The LGV originated from the angle between the PV and the SVs. **Type 4:** The LGV originated from the left branch of the PV.	Origin	Understanding the LGV anatomy is crucial before percutaneous transhepatic embolization of varicose veins or devascularization of the upper gastric region and abdominal esophagus. Moreover, in cases of esophageal variceal bleeding due to liver cirrhosis, LGV size indicates potential bleeding severity.
Lee & Lee et al.	2018	**Type I:** the LGV crossed the CHA and drained into the portal venous system (PVS). **Type II:** the LGV drained anteriorly to the CA **Type III:** The LGV crossed the SA and drained. **Types I and III** were further subdivided into anterior (a) and posterior (p) relative to CHA or SA. **Type IV:** the LGV drained directly into the liver parenchyma or the proximal PV near the PV bifurcation.	Course	Studying LGV variations before laparoscopic gastrectomy is crucial for preventing vein damage and preparing for potential hemorrhage.
Kuwada et al.	2015	The variant consisted of an ALGV and an ALHA directly entering the lateral segment of the liver via the hepatogastric ligament. The ALGV was divided at its entry point into the liver.	Drain	For curative lymph node dissection in gastric cancer, it is standard to cut the LGA and LGV at their origins. However, finding rare anomalies like concurrent ALGV and ALHA requires careful clinical consideration.
Ishigami et al.	2004	Large LGV contiguous to the superior lateral branch of the LPV through the gastrohepatic ligament, consistent with an ALGV. This aberrant vein is also connected to the posterior part of the umbilical portion of the LPV.	Drain	A retrospective analysis shows ALGV’s role as a decompression pathway, potentially reducing the severity of extensive variceal bleeding. This underscores the complexity of varicose vein pathophysiology and the need to evaluate anatomical abnormalities and their functional impact in treating portal hypertension and its complications.
Frey et al.	2022	**Case 1:** ALGV, which ends its course in the segment II parenchyma, corresponds to type 1 of the Unal classification, where it acts as a pure aberrant portal vein branching into liver sinusoids. **Case 2:** ALGV gives branches throughout the parenchyma and ends its course into the LPV, corresponding to type 2 of the Unal classification. **Case 3:** A large and single LGV is contiguous to the left branch of the portal vein, consistent with type 3 ALGV of the Unal classification.	Drain	ALGV serves as a hepatofugal collateral pathway in patients with advanced cirrhosis and portal hypertension. It also enables direct periportal spread of gastric tumors, affecting survival rates, especially in gastric cancer with venous invasion. Additionally, ALGV poses a risk of accidental hemorrhage during left gastric or hepatic surgeries.
Deneve et al.	2003	The LGV ascended from the lesser curvature and entered the left part of the porta hepatis. Similarly, the RGV ascended from the lesser curvature, passing in front of the common bile duct, and entered the porta hepatis directly. Both veins terminated in the intrahepatic segment of the LPV.	Drain	When the HPV becomes thrombosed, these variants may help maintain sufficient hepatopetal flow. As Bezzi suggests, they may be the only route for stenting a TIPS.
Caty et al.	2004	ARGV drains directly into the liver. While the LGV empties into the left aspect of the PV, the right one was observed to ascend from the lesser curvature of the stomach along the anterior right aspect of the common bile duct, directly reaching the porta hepatis.	Drain	The ARGV drains into the liver rather than the PV, potentially causing focal fatty infiltration and sparing in fatty liver disease. Imaging techniques, such as CT and ultrasound, can detect these anomalies, presenting as pseudolesions in the liver.
Choi et al.	2020	** Type 1:** ARGV was observed in 31 patients (1.5%). **Type 2:** ALGV was observed in 21 patients (1.0%).	Drain	Segmental liver atrophy can create a benign pseudotumor, complicating diagnosis and misguiding care, especially for cancer patients. Furthermore, the area where the AGV drains may show focal sparing in fatty livers, focal fat deposition, or hyperplastic changes, mimicking hepatic tumors in imaging. Pseudolesions are often found in this area, influenced by different metabolic, toxic, and hormonal environments from varied venous blood supplies.
Arhire et al.	2023	** Case 1: ** The ARGV runs about 6 cm, curving backward to align with the left portal branch. Inside the venous ligament’s fissure, it extends towards the third liver segment, branching into small parenchymal vessels. **Case 2:** The ARGV runs approximately 6 cm in an anteroposterior direction, positioning itself anteromedial to the left branch of the portal vein. It then enters the third liver segment. In both cases, pseudolesions appeared as diffuse, homogeneous hyperdense areas with unclear borders at the II and III liver segment boundary, surrounded by hypodense, fatty hepatic parenchyma.	Drain	The ARGV may produce a pseudolesion due to hepatic blood inflow mismatch (third inflow and transient hepatic attenuation difference-related hemodynamic mechanisms) or an associated underlying metabolic cause, such as a focal fat-sparing area within diffuse hepatic fatty infiltration. These pseudolesions may mimic liver tumors, so it is vital to search for such an aberrant vessel to rule out other diagnoses.
Mittal et al.	2015	**Type 1**: ALGV joins directly to the left branch of the portal vein instead of draining into the main portal trunk.	Drain	The aberrant vein draining into the LPV instead of the main PV leads to isolated left hepatic portal venous gas, resulting in gastric pneumatosis and an incarcerated hiatal hernia.
Miyaki et al.	1987	**Type 1**: The vein enters the liver directly from the left side of the hilus. **Type 2:** The vein collects several branches from the lesser curvature.	Course	To understand the unusual course of the LGV in humans and to clarify whether the LGV originates from the omphalomesenteric or subintestinal vein.
Ohkubo et al.	2000	**Type 1**: The LGV originating from the lesser curvature of the stomach runs along the hepatic branch of the vagus nerve through the lesser omentum to reach the hepatic hilus and directly enters the liver.	Drain	Understanding anatomical knowledge of interportal venous communication is essential for properly treating bleeding esophageal varices or performing angiographic embolization.
Alfaro et al.	2023	** Type 1:** Aberrant RGV drainage towards the liver causes a hypodense hepatic pseudolesion (HPS) located in hepatic segment IVb. **Type 2:** The LGV drains into the posterior margin of hepatic segment III, consistent with an ALGV causing a HPS localized to hepatic segments II and III. **Type 3:** The LGV drains into the posterior margin of hepatic segment III, consistent with an ALGV, and causes a small HPS localized to hepatic segment III.	Drain	AGVs may serve as an alternative venous drainage route in hypertensive gastropathy in cirrhotic patients or as a direct pathway for metastatic gastric cancer to the left liver. This variation can cause accidental bleeding during gastric or hepatic surgery, increasing surgery time and morbidity. Identifying AGVs on imaging is crucial to avoid misdiagnosis as liver lesions, which may lead to unnecessary procedures.
Seong et al.	2012	** Type 1:** Smooth continuation as a single channel into the peripheral portal vein. **Type 2:** Collateral connection into the peripheral portal vein. 2a: Single collateral channel. 2b: Multiple collateral channels. **Type 3:** Superficial parenchymal blush formation in small areas without demonstrable portal branches. 3a: unifocal. 3b: multifocal. **Type 4:** Network formation around the sectional or segmental portal vein.	Drain	Aberrant gastric venous drainage is crucial due to pseudolesion formation in the portal phase of CT angiography, highlighting cavernous transformation in main portal thrombosis, and unexpected hemorrhage during hepatobiliary surgery from a missed aberrant gastric venous drainage. The ARGV may also serve as a direct metastatic pathway for gastric cancer in the lesser curvature and a potential route for hepatofugal arterioportal shunt in main portal vein tumor thrombosis. Additionally, it can serve as an alternative for stent placement in a transjugular intrahepatic portosystemic shunt with main portal thrombosis.
Unal et al.	2015	** Type 1: ** The vein functions as a pure aberrant portal vein, branching out and flowing through the sinusoids. **Type 2:** The vein exhibits a parenchymatous distribution with anastomosis to the portal vein. **Type 3:** The vein follows a more cranial course, connecting to intrahepatic portal vein branches.	Drain	ALGV in gastric cancer patients can cause tumor spread to the liver. ALGV-related pseudolesions affecting the posterior segments II and III can mimic metastases; therefore, MRI can differentiate between pseudolesions and true lesions.
Unal et al.	2018	**Type 1**: short gastric vein invaded. Patients survived 6, 7, and 256 days. **Type 2:** gastric vein invaded. Patients survived 60 and 439 days. **Type 3:** ALGV invaded. Patients survived 105 and 187 days. **Type 4:** SMV via the right gastroepiploic vein invaded. Patients survived 120 and 1275 days. **Type 5:** Portal vein via the gastric vein. Patients survived 537 days (second patient N/ac).	Drain	Evaluate the value of CT-based diagnosis for venous invasion in gastric cancer patients, noting that survival rates are significantly low for those with ALGV and short gastric vein invasion. Thus, ALGV or short gastric vein invasion on CT may indicate a poor prognosis.
Wu et al.	2016	** Type 1:** LGV runs dorsal to the common hepatic artery. **Type 2:** LGV runs ventral to SA. **Type 3:** LGV runs between the common hepatic arteries and the SA. **Type 4:** LGV runs dorsal to SA. **Type 5:** LGV runs cranially into the LPV or hepatic parenchyma. **Type 6:** LGV runs ventral to the common hepatic artery. **Type 7:** Arterial variations impairing the reference frame function of the common hepatic artery and SA.	Variations of course	Development of a new classification system for variations of LGV. This system may help in the scientific description of LGV variations and facilitate the diagnosis of gastric cancer.
Yamagami et al.	1999	**Type 1**: Right gastric vein drains directly to the left lobe of the liver parenchyma around the falciform ligament.	Drain	The gastric vein right draining into the liver parenchyma around the falciform ligament may influence non-tumor abnormalities seen on CT angiography.
Muñoz & Fraum et al.	2023	**Type 1:** Right gastric vein directly perfuses a part of hepatic segment IV with intense uptake of the radiotracer in PSMA-PET/CT	Drain	Additional vascular supply to portions of the hepatic parenchyma may result in differential enhancement patterns on dynamic post-contrast imaging studies. ARGV represents an important consideration when assessing for metastatic disease because variants in hepatic vasculature can sometimes be mistaken for more serious pathologies.
Bezzi et al.	1995	To maintain flow, anomalous anastomoses between the RGV and the right or left portal vein branches were preserved.	Drain	Both cases reported are of a transjugular intrahepatic portosystemic shunt where the right gastric vein drains into branches of the portal vein and supplies its flow when a thrombosis occurs in the portal vein.
Deger & Bozer et al.	2023	ALGV	Drain	The ALGV causes pseudolesions in segments 2 and 3 of the liver parenchyma.
Gabata et al.	1997	An AGV ascended within the hepatoduodenal ligament, anterior to the main portal vein, reaching the porta hepatis. It then directly drained into the focal spared area at the posterior edge of segment IV.	Drain associated with fatty liver.	In cases of preserved focal area in the posterior border of segment IV in fatty liver, presents direct drainage to the liver in the AGV.
Hiwatashi et al.	1999	An AGV drains into segment II of the liver.	Drain associates a pseudolesion	In patients with metastasis data in the posterior lobes of the liver, as observed on CT, ultrasound, and venous angiography, an ALGV was identified that drained into segment II of the liver, generating pseudolesions that mimicked metastasis.
Matsui et al.	1994	The ARGV drains into segment IV of the liver.	Drain associates a pseudolesion.	17 patients out of 22 had a vein coming from the pylorus, the right gastric vein, with aberrant drainage directly into segment IV of the liver. 6 of these patients had direct intrahepatic drainage into a portal branch.
Matsui, Kadoya, Yoshikawa, et al.	1995	The ARGV drains into segments IV and I of the liver.	Drain	17/18 patients had aberrant drainage of the right gastric vein in the posterior aspect of segment 4 of the liver, while only one patient had aberrant drainage in segment 1.
Matsui et al.	1995	The ARGV drains into the area of focal preservation.	Drain	Aberrant drainage of the direct right gastric vein in focal areas of the liver in patients with fatty liver.
Natsume et al.	2010	52/126 LCV flowed into the portal vein 44/126 LCV flowed into the splenic vein, 22/126 flowed into the junction of these two veins	Drain	Utilizing various imaging techniques is essential for the successful execution of critical surgical procedures, as well as understanding the potential variations among patients.
Terayama et al.	2004	ALGV runs along the hepatogastric ligament toward the left side of the hepatic hilus, enters the II segment in the liver, and joins the intrahepatic portal venous branch. The corresponding area was focally spared of fatty liver.	Drain	LGV causes pseudolesions in segments II and IV of the liver on CT during arterial portography, and it may reveal a perfusion defect in the corresponding area.
Roi et al.	1993	**Type 1:** LGV drains in the portal vein (PV) **Type 2:** LGV drains in the splenoportal junction. **Type 3:** LGV drain in the splenic vein (SV) **Type 1A:** anterior LGV to the PV. **Type 1P:** posterior LGV to PV. **Type 2A:** anterior LGV to splenoportal junction. **Type 2P:** posterior LGV to splenoportal junction. **Type 3A:** anterior LGV to SV **Type 3P:** posterior LGV to SV.	Drain and course	Sonography of the termination of the LGV, despite the highly variable drainage site, enables an easy and accurate definition of its anatomical relationship with the adjacent vessels.
Rebibo et al.	2012	**Type A:** termination on the portal vein (PV) **Type B:** termination on the splenomesenteric trunk (SMT) **Type C:** termination on the splenic vein (SV)	Drain	Understanding the distribution and drainage area of the gastric vein is crucial, particularly during surgical resections of the pancreas, to avoid impacting the stomach’s drainage. This can be achieved through imaging techniques.

**Table 3:** Prevalence of readable articles.

Author	Year	Total, *n*	Prevalence (%)
Miyaki et al.	1987	245	2
Matsui, Kadoya, Yoshikawa, et al.	1995	122	18
Matsui et al.	1995	18	1
Seong et al.	2012	100	7
Rebibo et al.	2012	86	26
Li et al.	2016	234	43
Wu et al.	2016	805	77
Unal et al.	2018	530	11
Lee & Lee et al.	2018	405	12
Ünal & Karcaaltincaba et al.	2019	43,679	32
Choi et al.	2020	2021	52

**Table 4:** Subgroup analysis of the studies included in the systematic review analysis.

Parameters	Number of Studies and Subjects	Prevalence (%)	95% CI	I^2^	*p*-Value
Overall	11 (48,245)	8.32%	3.12–13.17	98.92%	-
Cadaveric	1 (245)	0.082%	-	-	*p* = 0.0001
Imaging	10 (48,000)	8.18%	3.49–12.91	89.11%	
Asia	8 (3950)	6.37%	4.19–8.98	88.12%	*p* = 0.032
Africa	0	-	-	-	
Europe	3 (44,295)	2.11%	0.77–3.99	91.35%	
America	0	-	-	-	
Oceania	0	-	-	-	
Male	7 (2552)	5.29%	3.12–9.33	77.11%	*p* = 0.024
Female	7 (1173)	2.43%	1.10–4.13	84.12%	
Gastric vein left	10 (48,000)	8.18%	7.01–10.12	77.10%	*p* = 0.012
Gastric veinright	3 (459)	3.29%	2.21–4.13	88.12%	

**Table 5:** Summary of clinical considerations (LGV—left gastric vein).

Author(s)	Year	Type of Consideration: Pathological, Surgical, Advantageous, Imaging	Description
Miyaki et al.	1987	Imaging	To determine the aberrant course of the LGV in humans by imaging and to elucidate whether the LGV derives from the omphalomesenteric vein or the subintestinal vein
Roi et al.	1993	Imaging	Ultrasonography of the LGV termination, despite significant variability in the drainage site, enables an easy and precise definition of its anatomical relationship with the adjacent vessels.
Matsui et al.	1994	Surgical	Segment IV receives a large blood supply; therefore, in the event of surgery in that area, it is necessary to check for any GV variant.
Bezzi et al.	1995	Surgical/Advantageous	In cases of hypertension of the portal vein system, if it is not possible to cannulate and the patient has an anomalous insertion of the RGV in the branches of the HPV system, the RGV can be cannulated to relieve portal hypertension
Matsui et al.	1995	Not indicated	Not indicated
Matsui, Kadoya, Yoshikawa, et al.	1995	Pathological	Relationship between the appearance of posterior masses in segment IV of a fatty liver and the aberrant drainage of the RGV in the same segment.
Gabata et al.	1997	Pathological	Focal preserved area at the posterior border of segment IV in fatty liver, showing direct drainage to the liver through the AGV
Yamagami et al.	1999	Imaging	The RGV draining into the hepatic parenchyma around the falciform ligament may influence the appearance of non-tumor abnormalities on CTA
Hiwatashi et al.	1999	Pathological	The relevance of using imaging and angiography to detect variations in GV drainage in case of pseudolesions, and thus avoid invasive procedures
Ohkubo et al.	2000	Surgical	Anatomical knowledge of interportal venous communication is essential to treat bleeding esophageal varices or angiographic embolization adequately.
Deneve et al.	2003	Advantageous	When the main HPV is thrombosed, ALGV and ARGV can contribute to maintaining sufficient hepatic flow and can be used as the sole route for stenting a TIPS
Ishigami et al.	2004	Advantageous	ALGV has a crucial role as a decompression pathway, possibly reducing the severity of extensive variceal bleeding
Caty et al.	2004	Pathological	The ARGV, by draining directly into the liver rather than the HPV, may contribute to focal fatty infiltration and focal sparing in fatty liver
Terayama et al.	2004	Pathological	LGV causes pseudolesions in segments II and IV of the liver, which are visible on a CT scan during arterial portography, and may reveal a perfusion defect in the corresponding area.
Natsume et al.	2010	Imaging	3DCTA is a valuable and essential modality for visualizing the precise anatomy surrounding the stomach preoperatively and for conducting safe operations.
Seong et al.	2012	Pathological	Aberrant gastric venous drainage is crucial for radiologists and clinicians due to the formation of pseudolesions from cavernous transformation in main hepatic vein thrombosis, and unexpected hemorrhage during hepatobiliary surgery from unidentified AGVs. The ARGV may also serve as a direct metastatic route for gastric cancer on the lesser curvature and an arterioportal bypass in tumor thrombosis of the main HPV.
Rebibo et al.	2012	Surgical	Preoperative analysis of the LGV is valuable because the vein can be identified in every case. Understanding the anatomical location of the termination enables subsequent resection to be initiated in a low-risk area.
Kuwada et al.	2015	Surgical	In the setting of curative lymph node dissection for gastric cancer, identifying aberrant LGV and ALHA requires careful clinical consideration. In such cases, the approach involved dividing the ALGV at its root while selectively preserving the ALHA
Mittal et al.	2015	Pathological	The aberrant vein draining into the LPV instead of the main HPV results in isolated left hepatic venous portal gas, resulting in gastric pneumatosis and an incarcerated hiatal hernia
Unal et al.	2015	Pathological	The ALGV in patients with gastric cancer may lead to the direct spread of the tumor to the liver. Hepatic pseudolesions associated with ALGV affecting the posterior aspect of liver segments II and III may mimic metastasis
Li et al.	2016	Pathological	Variceal bleeding is a complication in individuals with portal hypertension, with the LGV being the primary source of blood supply to esophagogastric varices. Furthermore, esophageal variceal bleeding occurs more frequently in patients with an enlarged LGV diameter exceeding 5–6 mm, a parameter indicative of portal hypertension
Wu et al.	2016	Not indicated	Not indicated
Lee & Lee et al.	2018	Surgical	Type II (Lee classification) LGV has a relatively lower risk of injury during laparoscopic gastrectomy; there is no need to perform a complete lymph node dissection around the CA
Unal et al.	2018	Pathological	The ALGV in a patient with gastric cancer may indicate a worse prognosis
Ünal & Karcaaltincaba et al.	2019	Advantageous	The presence of the ALGV type III variant maintained the flow of the LPV in patients with main HPV thrombosis
Choi et al.	2020	Pathological	The segment drained by the AGV may exhibit focal sparing in fatty livers, focal fat deposition, or hyperplastic changes, which can mimic liver tumors on imaging studies. Segment II atrophy was found more frequently in patients with AGVs
Frey et al.	2022	Pathological	Segment II atrophy is observed in 33.3% of patients with ALGV. ALGV provides a direct route for periportal dissemination of the gastric tumor
Arhire et al.	2023	Pathological	ARGV can produce pseudoinjury due to a mismatch in hepatic blood flow (hemodynamic mechanisms related to the third flow and the transient hepatic attenuation difference), but also due to an associated and underlying metabolic cause
Alfaro et al.	2023	Advantageous/Pathological	AGVs could act as an alternative route for venous drainage in hypertensive gastropathy in cirrhotic patients or as a direct metastatic route for gastric cancer on the left side of the liver
Deger & Bozer et al.	2023	Imaging	The importance of recognizing vascular anomalies, such as ALGV, in the liver vasculature and linking them to pseudolesions in the liver parenchyma is crucial for avoiding invasive procedures.
Muñoz & Fraum et al.	2023	Imaging	ARGV represents a crucial consideration when evaluating metastatic disease, as variants in the hepatic vasculature can sometimes be misdiagnosed as more severe pathologies.

**Table 6:** Summary subgroups: imaging, cadaver, or in vivo (surgery).

Author(s)	Year	Sample n	Study Type Imaging, Cadaveric, or Surgery
Miyaki et al.	1987	245	Cadaveric
Roi et al.	1993	86	Imaging
Matsui et al.	1994	122	imaging
Bezzi et al.	1995	2	imaging
Matsui et al.	1995	18	imaging
Matsui, Kadoya, Yoshikawa, et al.	1995	17	imaging
Gabata et al.	1997	17	imaging
Yamagami et al.	1999	1	Imaging
Hiwatashi et al.	1999	1	imaging
Ohkubo et al.	2000	1	Cadaveric
Deneve et al.	2003	1	Cadaveric
Ishigami et al.	2004	1	imaging
Caty et al.	2004	1	Cadaveric
Ishigami et al.	2004	1	imaging
Terayama et al.	2004	1	Imaging
Natsume et al.	2010	126	imaging
Seong et al.	2012	100	Imaging
Rebibo et al.	2012	86	imaging
Kuwada et al.	2015	1	imaging
Mittal et al.	2015	1	Cadaveric
Unal et al.	2015	not specified	not specified
Li et al.	2016	234	Imaging
Wu et al.	2016	805	Imaging
Lee & Lee	2018	405	in vivo
Unal et al.	2018	530	Imaging
Ünal & Karcaaltincaba et al.	2019	43,679	Imaging
Choi et al.	2020	2021	Cadaveric
Frey et al.	2022	3	imaging
Arhire et al.	2023	2	Cadaveric
Alfaro et al.	2023	3	Imaging
Deger & Bozer et al.	2023	1	imaging
Muñoz & Fraum et al.	2023	1	imaging

## Supplementary Materials

The following supporting information can be downloaded at: https://www.mdpi.com/article/10.3390/jcm14113630/s1, Table S1: Details of the search strategy. Table S2. Excluded studies and the reasons for their exclusion.

## Abbreviations

HPV: hepatic portal vein, LGV: left gastric vein, RGV: right gastric vein, CA: celiac axis, SV: splenic vein, ALGV: aberrant left gastric vein, LPV: left portal vein, CHA: common hepatic artery, SA: splenic artery, ARGV: aberrant right gastric vein, TIPS: transjugular intrahepatic portosystemic shunt, ALHA: aberrant left hepatic artery, RGA: right gastric artery, and LGA: left gastric artery.
