# Supplementary material for: The Gastric Vein Variants: An Evidence-Based Systematic Review of Prevalence and Clinical Considerations
Source: J Clin Med. 2025 May 22;14(11):3630. doi: 10.3390/jcm14113630 (PMC12155747; doi:10.3390/jcm14113630)
Supplement: Supplementary file 1 [file jcm-14-03630-s001.zip › jcm-3612887-supplementary.pdf]

Supplementary Table S1. Details of the search strategy.

Database	Search strategy	Results	
		**10-01-25**	**01-05-2025**
Medline	Gastric veins variants OR gastric veins aberrant OR gastric veins anomalies AND clinical anatomy Filters: English, Humans ((((“gastrics”[All Fields] OR “stomach”[MeSH Terms] OR “stomach”[All Fields] OR “gastric”[All Fields]) AND (“vein s”[All Fields] OR “veins”[MeSH Terms] OR “veins”[All Fields]) AND (“variant”[All Fields] OR “variant s”[All Fields] OR “variants”[All Fields])) OR ((“gastrics”[All Fields] OR “stomach”[MeSH Terms] OR “stomach”[All Fields] OR “gastric”[All Fields]) AND (“vein s”[All Fields] OR “veins”[MeSH Terms] OR “veins”[All Fields]) AND (“aberrance”[All Fields] OR “aberrances”[All Fields] OR “aberrancies”[All Fields] OR “aberrancy”[All Fields] OR “aberrant”[All Fields] OR “aberrants”[All Fields] OR “aberrated”[All Fields] OR “aberrating”[All Fields] OR “aberration”[All Fields] OR “aberrational”[All Fields] OR “aberrations”[All Fields] OR “aberrator”[All Fields] OR “aberrators”[All Fields])) OR ((“gastrics”[All Fields] OR “stomach”[MeSH Terms] OR “stomach”[All Fields] OR “gastric”[All Fields]) AND (“vein s”[All Fields] OR “veins”[MeSH Terms] OR “veins”[All Fields]) AND (“abnormalities”[MeSH Subheading] OR “abnormalities”[All Fields] OR “anomalies”[All Fields] OR “anomalie”[All Fields] OR “anomaly”[All Fields]))) AND (“clin anat”[Journal] OR (“clinical”[All Fields] AND “anatomy”[All Fields]) OR “clinical anatomy”[All Fields])) AND ((humans[Filter]) AND (english[Filter]))	15	16
Wos	Gastric veins variants OR gastric veins aberrant OR gastric veins anomalies AND clinical anatomy	70	70
CINAHL	Gastric veins variants OR gastric veins aberrant OR gastric veins anomalies AND clinical anatomy	9	9

SCOPUS	Gastric veins variants OR gastric veins aberrant OR gastric veins anomalies AND clinical anatomy	110	110
Google scholar	Gastric veins variants OR gastric veins aberrant OR gastric veins anomalies AND clinical anatomy	20	20
EMBASE	Gastric veins variants OR gastric veins aberrant OR gastric veins anomalies AND clinical anatomy	53	53
	Total	275	279

* All searches were carried out on April, 2025.

Supplementary Table S2. Excluded studies and the reasons for their exclusion.

N°	Reference	Reason
1	Elzeneini W, Woodward C, Shalaby MS. Neonatal gastric perforation: when to expect and how to manage. Br J Hosp Med (Lond). 2019 Mar 2;80(3):i. doi: 10.12968/hmed.2019.80.3.i. PMID: 30860906.	Pathological alteration, not attributable to an anatomical variant
2	Shigeta T, Yamauchi Y, Oda A, Tachibana S, Hirao T, Nakamura R, Yoshida H, Okishige K, Goya M, Sasano T. Prevalence of gastric hypomotility after additional cryoballoon ablation of the left atrial roof. Pacing Clin Electrophysiol. 2022 Jan;45(1):5-13. doi: 10.1111/pace.14387. Epub 2021 Oct 31. PMID: 34679229.	Pathological alteration, not attributable to an anatomical variant
3	Szabó G, Benyó I, Sándor J. The effect of haemorrhage on gastric circulation and acid output in the Injury. 1979 Feb;10(3):190-3. doi: 10.1016/0020-1383(79)90006-8. PMID: 310421.	Surgical alteration, not attributable to an anatomical variant
4	Kobayashi H. Estrogen synthesis in gastric parietal cells and secretion into portal vein. Anat Sci Int. 2020 Jan;95(1):22-30. doi: 10.1007/s12565-019-00510-5. Epub 2019 Nov 16. PMID: 31734841.	Other vein abdominal region
5	Yuasa Y, Okitsu H, Goto M, Kuramoto S, Tomibayashi A, Matsumoto D, Edagawa H, Mori O, Tani R, Akagawa T, Kinoshita M, Akagawa Y, Tani H, Ohnishi N, Shirono R. Three-dimensional CT for preoperative detection of the left gastric artery and left gastric vein in laparoscopy-assisted distal gastrectomy. Asian J Endosc Surg. 2016 Aug;9(3):179-85. doi:	Surgical alteration, not attributable to an anatomical variant
6	Hackert T, Weitz J, Büchler MW. Reinsertion of the gastric coronary vein to avoid venous gastric congestion in pancreatic surgery. HPB (Oxford). 2015 Apr;17(4):368-70. doi: 10.1111/hpb.12321. Epub 2014 Jul 24. PMID: 25059096; PMCID: PMC4368403.	Surgical alteration, not attributable to an anatomical variant

7	White RN, Parry AT. Morphology of congenital portosystemic shunts involving the right gastric vein in dogs. J Small Anim Pract. 2015 Jul;56(7):430-40. doi: 10.1111/jsap.12355. Epub 2015 Apr 14. PMID: 25871881.	Example animals
8	Zou X, Liu L, Tan F, Tang H, Hu D, Li Z, Wang Q, Shen Y. Non-contrast-enhanced MR angiography of left gastric vein in patients with gastroesophageal varices: morphology and blood supply analysis. Eur Radiol. 2024 Jul;34(7):4686-4696. doi: 10.1007/s00330-023-10497-3. Epub 2023 Dec 22. PMID: 38133674.	Pathological alteration, not attributable to an anatomical variant
9	Takayasu K, Aoki K, Ichikawa T, Ohmura T, Sekiguchi R, Terauchi T, Takayama T. Aberrant right gastric vein directly communicating with left portal vein system. Incidence and implications. Acta Radiol. 1990 Nov;31(6):575-7. PMID: 2278780.	Other vein abdominal region
10	White RN, Parry AT. Morphology of congenital portosystemic shunts emanating from the left gastric vein in dogs and cats. J Small Anim Pract. 2013 Sep;54(9):459-67. doi: 10.1111/jsap.12116. Epub 2013 Jul 25. PMID: 23888909.	Example animals
11	Matsutani S, Furuse J, Ishii H, Mizumoto H, Kimura K, Ohto M. Hemodynamics of the left gastric vein in portal hypertension. Gastroenterology. 1993 Aug;105(2):513-8. doi: 10.1016/0016-5085(93)90728-u. PMID: 8335205.	Pathological alteration, not attributable to an anatomical variant
12	Oomatia A, Carroll N, Safranek PM. Acute gastric necrosis and air in the hepatic-portal vein secondary to a strangulated paraumbilical hernia. Lancet. 2013 Nov 23;382(9906):1733. doi: 10.1016/S0140-6736(12)61778-4. Epub 2013 May 10. PMID: 23664059.	Pathological alteration, not attributable to an anatomical variant
13	Sandroussi C, McGilvray ID. Gastric venous reconstruction after radical pancreatic surgery: case report and review of the literature. J Gastrointest Surg. 2010 Jun;14(6):1027-30. doi: 10.1007/s11605-010-1192-0. Epub 2010 Apr 13. PMID: 20387128.	Pathological alteration, not attributable to an anatomical variant
14	Muhletaler C, Gerlock AJ Jr, Goncharenko V, Avant GR, Flexner JM. Gastric varices secondary to splenic vein occlusion: radiographic diagnosis and clinical significance. Radiology. 1979 Sep;132(3):593-8. doi: 10.1148/132.3.593. PMID: 472232.	Pathological alteration, not attributable to an anatomical variant
